# Epinephrine affects motility, and increases adhesion, biofilm and virulence of *Pseudomonas aeruginosa* H103

**DOI:** 10.1038/s41598-019-56666-7

**Published:** 2019-12-27

**Authors:** Mélyssa Cambronel, Damien Tortuel, Kelly Biaggini, Olivier Maillot, Laure Taupin, Karine Réhel, Isabelle Rincé, Cécile Muller, Julie Hardouin, Marc Feuilloley, Sophie Rodrigues, Nathalie Connil

**Affiliations:** 1 Laboratoire de Microbiologie Signaux et Microenvironnement (LMSM) EA 4312, Normandie Université – Université de Rouen, Évreux, 27000 Evreux, France; 2 Laboratoire de Biotechnologie et Chimie Marines (LBCM) EA 3884, IUEM, Université de Bretagne-Sud, 56100 Lorient, France; 3 Unité de Recherche Risques Microbiens (U2RM), EA 4655, UFR des sciences, Normandie Université, Université de Caen, 14000 Caen, France; 4 Laboratoire Polymères, Biopolymères, Surfaces, UMR 6270 CNRS, Plateforme Protéomique, PISSARO, Normandie Université, Université de Rouen, 76130 Mont Saint Aignan, France

**Keywords:** Biofilms, Pathogens

## Abstract

Microbial endocrinology has demonstrated for more than two decades, that eukaryotic substances (hormones, neurotransmitters, molecules of the immune system) can modulate the physiological behavior of bacteria. Among them, the hormones/neurotransmitters, epinephrine (Epi) and norepinephrine (NE), released in case of stress, physical effort or used in medical treatment, were shown to be able to modify biofilm formation in various bacterial species. In the present study, we have evaluated the effect of Epi on motility, adhesion, biofilm formation and virulence of *Pseudomonas aeruginosa*, a bacterium linked to many hospital-acquired infections, and responsible for chronic infection in immunocompromised patients including persons suffering from cystic fibrosis. The results showed that Epi increased adhesion and biofilm formation of *P. aeruginosa*, as well as its virulence towards the *Galleria mellonella* larvae *in vivo* model. Deciphering the sensor of this molecule in *P. aeruginosa* and the molecular mechanisms involved may help to find new strategies of treatment to fight against this bacterium.

## Introduction

*Pseudomonas aeruginosa* is a Gram-negative opportunistic pathogen known for its implication in many hospital-acquired infections and in immunocompromised patients suffering from cystic fibrosis^1^. Furthermore, it is a pathogen of concern, since it was classed as critical by the World Health Organization in 2017, due to its antibiotic resistance. The pathogenicity of this bacterium is associated with its capacity to induce chronic infections (strong biofilms, which are difficult to treat) and acute infections where many virulence factors can be secreted. *P. aeruginosa* possesses a large genome (6.3 million base pairs) including many regulatory two-component systems and transcriptional regulators which contribute to its remarkable environmental adaptability^2,3^.

During host infection, bacteria are exposed to many eukaryotic substances such as immune system molecules (interleukin), peptides hormones (CNP, substance P) and neurotransmitters (GABA)^4^. Molecules released under host stress can influence host-pathogens interactions and bacterial pathogenicity^5^. Stress, trauma^6,7^, physical effort and therapy utilization of inotropes leads to the presence of catecholamines in the human body, which gather three endogenous molecules: dopamine, norepinephrine (NE) and epinephrine (Epi). These substances were found to be able to modulate the growth of Gram-negative pathogens like *Escherichia coli*^8^, *Vibrio cholerae*^9^, *Helicobacter pylori*^10^, *Salmonella enterica* and *Yersinia enterocolitica*^11^. Furthermore, some bacteria increased their virulence when exposed to catecholamines such as *Vibrio harveyi*, which was more virulent towards brine shrimp larvae, following a treatment with NE or dopamine^12^. The motility and capacity to form biofilm of *Escherichia coli* O157:H7 and *V. harveyi* were also found to be enhanced in presence of catecholamines^12,13^. In *P. aeruginosa* PA14, NE modulates the virulence and motility of the bacterium, and the *las* quorum-sensing pathway may be involved in the response towards this molecule^14^. In another study conducted on *P. aeruginosa*, strain PAO1, the authors showed that NE was able to repress the production of siderophore and the expression of *toxA* coding an important virulence factor exotoxin A^15^, rather than enhancing virulence production as seen with other bacteria.

Contrary to NE, prior to this study, very little information was available concerning the behavior of *P. aeruginosa* exposed to Epi. In this context, the aim of our study was to investigate the effect of this molecule on *P. aeruginosa* motility, adhesion, biofilm formation and virulence.

## Results

### Motility of *P. aeruginosa* H103

**Figure 1:**
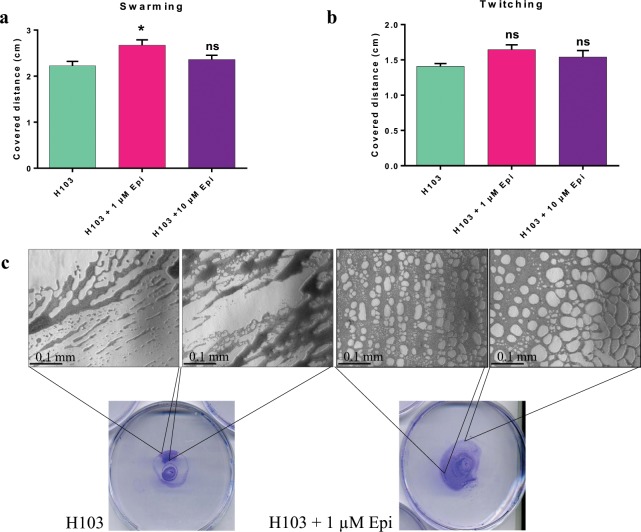
Effect of Epi on *P. aeruginosa* H103 motility. (**a**) Covered distance by swarming motility on LB 0.6% agar, after 24 h, (**b**) Covered distance by twitching motility on LB 1% agar, (**c**) Macroscopic and microscopic observations of twitching. Micrographs were taken with an Axio Vert A.1 inverted microscope, scale bar: 0.1 mm. The error bars indicate the standard error of the mean (SEM). Two-tailed paired *t*-test was used, (n = 6 and n = 3 for swarming and twitching assays respectively), *p = 0.0125, ns: not significant.

As motility was found to be modified in a large amount of Gram-negative bacteria after exposure to NE or Epi^13,16–19^, we decided to study this effect on *P. aeruginosa* H103 (Fig. 1). The results obtained showed that the radial growth of swarming phenotype was increased in presence of 1 µM Epi (Fig. 1a) and the twitching motility was qualitatively modulated (Fig. 1b). Indeed, the microscopic observation revealed that in presence of Epi, the twitching of *P. aeruginosa* H103 seemed to form a more well-organized network, compared to the untreated bacteria (Fig. 1c). As type IV pili can be involved in swarming and twitching of *P. aeruginosa* H103^20,21^, we then completed this study by using PO4 phage, which effect on *P. aeruginosa* H103 is highly dependent on type IV pili.

### PO4 phage sensitivity of *P. aeruginosa* H103

**Figure 2:**
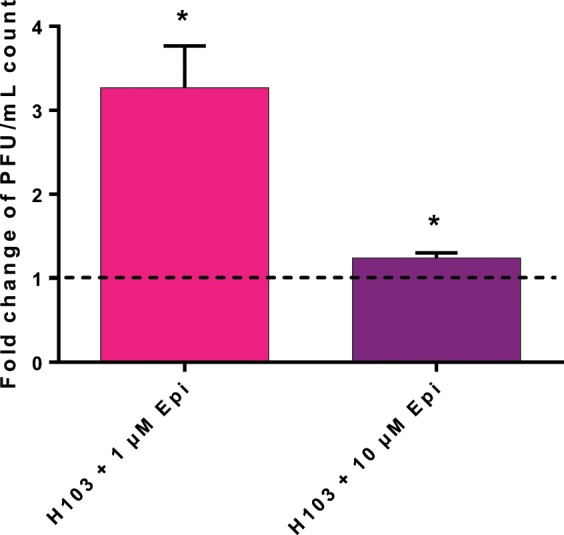
Effect of Epi on phage PO4 sensitivity. Phage PO4 sensitivity towards *P. aeruginosa* H103 was tested in presence of 1 and 10 µM of Epi after 24 h. Plaque lysis were enumerated and expressed in PFU/mL, fold change is expressed for each condition. The error bars indicate the standard error of the mean (SEM). Two-tailed paired *t*-test was used (n = 4), *p = 0.025.

Bacteriophages require a specific component of bacteria for host infection, like lipopolysaccharides for the phage PaP1^22^ or proteins on the bacterial surface. PO4 bacteriophages bind to the type IV pili of *P. aeruginosa*. To test the hypothesis of modulation of type IV pili in presence of Epi, a double agar overlay plaque assay was carried out. The results of this experiment, presented on Fig. 2, showed that *P. aeruginosa* H103 was more sensitive to the PO4 bacteriophage in presence of Epi. Indeed, the number of lysis plaque was higher when treated with 1 and 10 µM Epi, leading to 3.27-fold increase ± 0.5, and 1.24 ± 0.05, respectively. This means that when *P. aeruginosa* H103 was in contact with Epi, its sensitivity towards the PO4 phage was enhanced. This result reinforces the hypothesis that Epi may modify the type IV pili structure resulting in the modification of motility observed above.

### Adhesion of *P. aeruginosa* H103

**Figure 3:**
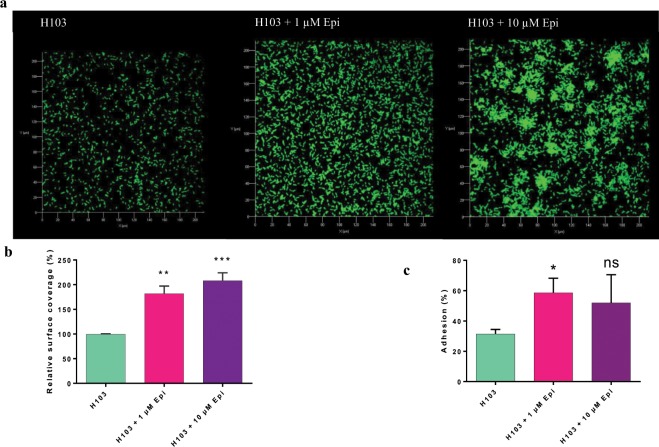
Effect of Epi on adhesion of *P. aeruginosa* H103. (**a**) Top view of *P. aeruginosa* H103-GFP adhesion observed by CLSM with two different concentrations of Epi (1 and 10 µM), after 2 h. (**b**) Image analysis by COMSTAT. Data were normalized according to the untreated control set at 100%. (**c**) Adhesion assay on Caco-2/TC7 cells, *p = 0.0418. The error bars indicate the standard error of the mean (SEM). Two-tailed paired *t*-test was used (n = 5), **p = 0.001222, ***p = 0.000413, ns: not significant.

Adhesion to a surface is the first step which allows bacteria to colonize an environment and thereafter, to form biofilm. Bacteria possess many components that help them to adhere on surfaces, like adhesin or the type IV pili. In this work, we investigated the effect of 1 and 10 µM Epi on adhesion of *P. aeruginosa* H103 to abiotic and biotic surfaces. The results obtained are presented on Fig. 3. A COMSTAT analysis showed that *P. aeruginosa* H103 displayed an increased surface coverage (181% ± 15%) when treated with 1 µM of Epi, compared to control (Fig. 3b). A higher concentration (10 µM of Epi) also increased the covered surface of *P. aeruginosa* H103 (207% ± 16%) (Fig. 3b). Moreover, *P. aeruginosa* H103 seems to form aggregates when treated with 10 µM of Epi (Fig. 3a).

The capacity of *P. aeruginosa* H103 to adhere on biotic surface was then evaluated on Caco-2/TC7 cell line after 2 h of contact (Fig. 3c). The results showed that 1 µM of Epi significantly enhanced the capacity of the bacteria to adhere to these cells (58% ± 9.6%) compared to control (31% ± 3%).

### Biofilm formation of *P. aeruginosa* H103

**Figure 4:**
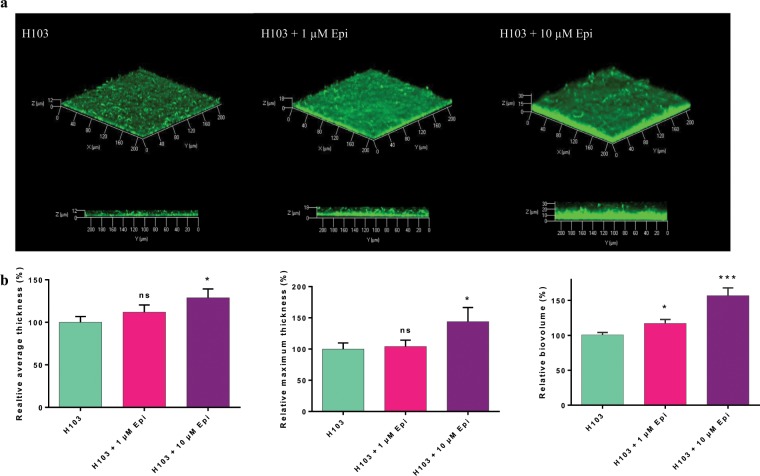
Effect of Epi on *P. aeruginosa* H103 biofilm formation. (**a**) Three dimensional (3D) and side views of *P. aeruginosa* H103-GFP 24 h biofilm observed by CLSM, in presence of 1 or 10 µM of Epi. (**b**) Images analysis by COMSTAT. Data were normalized according to the untreated control set at 100%. The error bars indicate the standard error of the mean (SEM) of at least three independent experiments. Two-tailed paired *t*-test was used (n = 4), *p < 0.05, ***p < 0.001, ns: not significant.

A flow-cell device with glass surface was used to investigate the biofilm formation of *P. aeruginosa* H103 in dynamic conditions to mimic *in vivo* conditions (Fig. 4). For both concentrations of Epi (1 and 10 µM) administrated continuously during biofilm formation, no modifications of biofilm architecture were observed compared to control (Fig. 4a). However, images analysis using COMSTAT software showed that average and maximum thicknesses were significantly increased in presence of 10 µM Epi compared to untreated biofilm, with +29% ± 10% (*p* = 0.0450) and +44% ± 22% (*p* = 0.0140), respectively. No significant difference of the average and maximum thicknesses were observed with 1 µM of Epi, but the biovolume reflecting the bacterial biomass was increased with +18% ± 5.7% (*p* = 0.0205) when exposed to 1 µM of this molecule and by +57% ± 11.4% (*p* < 0.0001) for 10 µM Epi (Fig. 4b).

### Virulence of *P. aeruginosa* H103 towards *G. mellonella*

**Figure 5:**
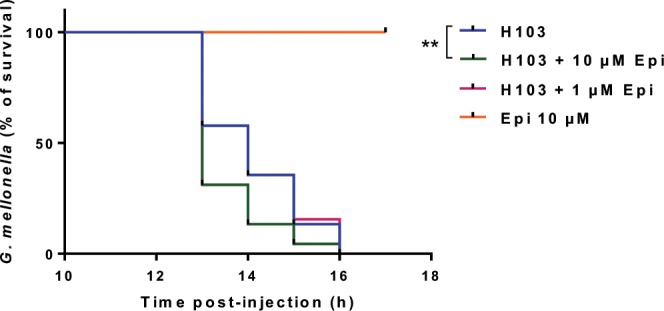
Virulence assay on *G. mellonella*. The virulence of *P. aeruginosa* H103 was tested towards the caterpillar *G. mellonella* larvae, during 17 h. Log-rank (Mantel-Cox) test was used, **p = 0.0086.

The pathogenicity of *P. aeruginosa* H103 treated or not with 1 or 10 µM Epi was assessed with the *in vivo* model *G. mellonella*, which has been developed in recent years, as an alternative to murine or other vertebrate models of infection to contribute to the 3Rs (reduction, replacement, and refinement) of animal use in scientific research. The results of this experiment, presented on Fig. 5, showed that the mortality of the larvae was accelerated when bacteria were treated and co-injected with 10 µM Epi. Indeed, only 31% of the larvae remained alive at 13 h post-infection in the 10 µM Epi treated conditions compared to 58% of survival for infection with the pathogen alone. On the contrary, a treatment with 1 µM Epi had no effect on pathogenicity of *P. aeruginosa* H103. A control negative was also used to be sure that Epi had no direct effect on larvae mortality.

## Discussion

Bacteria can be exposed to a multitude of host cell molecules such as catecholamines^7^ that could enhance the growth of many gram-negative bacteria^8,9,12,17,18,23–26^ as well as their virulence^19,27^. Till now, only few studies were conducted on the effect of these molecules on *P. aeruginosa*, which is an opportunistic pathogen, often found in hospital-acquired infections. Freestone and collaborators^28^ have shown that inotropes (dopamine, Epi and NE) are able to increase the biofilm formation of *P. aeruginosa*. Hedge *et al*.^14^ showed that NE increases *P. aeruginosa* PA14 virulence through the *las* quorum-sensing pathway by inducing, among others, the expression of *lasA* and *lasB*. Li *et al*.^15^ demonstrated that NE decreased siderophore production and *toxA* expression in PAO1.

Epi and catecholamines in general, can promote bacterial motility. In the present study, we showed that the swarming seemed to be slightly affected only at 1 µM Epi; moreover, the twitching motility displayed modifications in microscopic phenotype. In previous work, we have already observed that the swarming of *P. fluorescens* can also be affected by exposure to 1 µM Epi^29^. Twitching motility of *P. aeruginosa* PA14 strain was increased by Epi^28^. The present work showed that the stimulation of the twitching and the sensitivity to the PO4 bacteriophage are connected. Indeed, the PO4 phage needs the type IV pili to infect our strain^30^, and we saw that Epi enhance the intensity of the twitching, which indirectly measure the type IV pili function^31^. Production of rhamnolipids (another structure that can be involved in motility) has been quantified (Supplementary data 1) and no differences between treated and control conditions have been observed, reinforcing the hypothesis that the effect of Epi on motility may come from the type IV pili or the flagellum.

We demonstrated that upon exposure to Epi (1 and 10 µM), *P. aeruginosa* H103 adhesion on abiotic surface was increased and a concentration of 10 µM of Epi led to bacterial cell-cell attachment and microcolony formation. Lyte *et al*. (2003) demonstrated that adhesion of *Staphylococcus epidermidis* on polystyrene and silicon was increased upon addition to catecholamines inotropes (dopamine and NE)^32^. These authors also found the presence of bacterial aggregates following a microscopic evaluation of the biofilm. An enhancement of a specific biofilm exopolysaccharides production was proposed as an explanation for these phenomena. Attachment to surface was studied with *P. aeruginosa* PA14 on endotracheal tubes and the presence of cell-cell attachment was described as well^28^.

Biofilms enhance bacterial growth and survival by providing access to nutrients and provide protection from host immune system molecules and antimicrobial compounds. Furthermore, pathogenicity is often closely related to bacterial biofilm formation^33–35^. In the present study, we have examined the biofilm formation of *P. aeruginosa* under a continuous exposure (24 h) of Epi in dynamic conditions (i.e. flow cell system). We have observed an increased biovolume or thickness in presence of 1 or 10 µM of this molecule. None of these concentrations affected the growth of *P. aeruginosa* (Supplementary data 2). Freestone and collaborators (2012) also described an increased capacity of *P. aeruginosa* to form biofilm on eukaryotic cells and on endotracheal tube^28^ when treated with Epi on serum-SAPI medium, which mimics more closely the *in vivo* conditions. Other authors also showed an increase of biofilm formation when treated with catecholamines inotropes^12,13,32,36^.

The mechanism by which Epi could increase biofilm formation remains unclear. We can hypothesize that the impact of Epi on the type IV pili could contribute to the increase of adhesion and then biofilm formation. The type IV pili play an important role in the establishment of bacterial adhesion on abiotic surface (twitching motility) as well as in the development of microcolony and is required for biofilm formation by *P. aeruginosa*^37–40^.

The increasing ability of *P. aeruginosa* H103 to form biofilm when exposed to Epi suggested a role of this stress hormone in virulence. In our case, Epi at 10 µM significantly increased *P. aeruginosa* virulence towards *G. mellonella*. Similarly, Yang *et al*., (2014) demonstrated that catecholamines could enhance the virulence of pathogenic bacteria (*Vibrio harveyi)* towards a gnotobiotic brine shrimp larvae model^12^. Other authors studied the virulence of *Vibrio campbellii* BB120 toward giant freshwater prawn larvae, and showed that NE and dopamine were able to modulate the virulence of this bacterium^17^. In the prawn larvae model, the virulence can be increased by the production of extracellular protease^41^ and by the utilization of the type III secretion system^42^.

The interaction between bacteria and the host depends on their capacity to communicate. The production of catecholamines, which can occur after a trauma, stress or a physical effort, influences the bacterial pathogenicity^6^. Bacteria have adapted to their environment by developing various mechanisms, and two-component system is one of them. In *E. coli* O157:H7, two different systems, QseC/QseB and QseE/QseF have been described for their implication in sensing the presence of Epi or NE^43–45^. Other alternative adrenergic receptors have been found in organisms such as *S. Typhimurium* (BasS/R) or in *S. enterica* serovar Typhi^46^ (CpxA/R). To our knowledge, no adrenergic sensors have been identified in *P. aeruginosa* up to now. Investigations regarding the presence of homologous sequences to QseC in *P. aeruginosa* H103 have been performed in our laboratory. Some putative catecholamines receptors are now considered for further research. Deciphering the mechanism by which pathogenic bacteria are able to respond to host stress hormones could help understand how the signal is perceived and transduced, and the pathways impacted by these molecules. Proteomic analysis of *P. aeruginosa* H103 treated with Epi will also help us to discover how Epi acts on bacteria. *P. aeruginosa* can be present and responsible for skin, lung or intestine infections, where many eukaryotic substances are secreted; it is necessary to develop a way to prevent these phenomena to happen. Finding new therapeutic strategies could help to block bacterial capacity to perceive these eukaryotic signals and may reduce virulence.

## Materials and Methods

### Bacterial strains and growth conditions

The bacteria used in this study were *P. aeruginosa* H103 (PAO1 derivative strain)^47^ and the H103-GFP strain tagged with the green fluorescent protein (GFP)-encoding pHC60 plasmid (*gfp*, tet^R^)^48^. They were routinely grown at 37 °C under a rotary shaker (180 rpm) in Luria Bertani (LB) medium (containing 5 g/L NaCl). Depending on the assay, cultures were exposed to Epi (Sigma-Aldrich) from 1 to 10 µM. Tetracycline was used at 125 µg/mL for the culture of H103-GFP strain.

### Motility assays

Swarming and twitching motility were tested on LB with 0.6% and 1% (w/v) agar, respectively, and containing 1 µM or 10 µM as previously described^49,50^. Swarming plates were inoculated using a bacterial suspension spotted on the surface and incubated for 24 h at 37 °C. For twitching assay, the suspension was spotted underneath the agar layer. After 24 h at 37 °C, agar was removed, and cells attached to the Petri dish bottom were stained with crystal violet 0.4%. Micrographs were taken using a Zeiss Axio Vert A.1 inverted microscope (x40) (Zeiss, Germany) with a QImaging QIClick camera and Q-capture pro 7 software (QImaging, Canada). For both motility assays, each experiment was conducted three times with at least three biological replicates by test.

### PO4 phage sensitivity assay

Sensitivity of PO4 bacteriophage towards *P. aeruginosa* H103 was tested in 1.5% LB agar plate, with or without Epi. The double agar overlay method was used for the first step of phage isolation and later for precise titration of phage suspension^51^. Dilutions of phage suspension were mixed with 100 µl of *P. aeruginosa* H103 (10^8^ CFU/mL) in molted agar (supplemented or not with 1 µM or 10 µM Epi) and then poured on the top of LB agar plate. The plates were incubated at 37 °C for 24 h. Lysis plaques were then counted, and results were expressed in PFU/mL. Data were obtained from four independent experiments.

### Biofilm culture

Biofilms grown under hydrodynamic conditions were performed in a three channel flow cell (1 × 40 × 44 mm; Biocentrum, DTU, Danemark)^52^. The flow system was assembled, prepared and sterilized as described by Tolker-Nielsen and Sternberg^53^. The substratum consisted of a microscope glass coverslip (24 × 50 st1 [Knittel Glasser, Germany]). Each channel was inoculated with 250 µl of an overnight culture of *P. aeruginosa* H103 diluted to an OD_580_ of 0.1 (10^8^ CFU/mL) in physiological water (NaCl 0.9%). A two-hour attachment step was performed without any flow. A flow (2.5 mL/h) of LB medium with or without Epi (1 and 10 µM) was then applied for 24 h using a Watson Marlow 205U peristaltic pump (Watson Marlow, UK). The attached bacteria or biofilms were observed by confocal laser scanning microscopy (CLSM) as described below.

### Confocal laser scanning microscopy (CLSM)

Biofilm observations were performed with a Zeiss LSM710 (Zeiss, Germany) using a 40x oil immersion objective. Bacteria were detected by monitoring the GFP fluorescence. GFP was excited at 488 nm and fluorescence emission was detected between 500 and 550 nm. Images were taken every micrometer throughout the whole biofilm depth. For visualization and processing of three-dimensional (3D) image data, the Zen 2.1 software (Zeiss, Germany) was used. Quantitative analyses of image stacks were performed using the COMSTAT software (http://www.imageanalysis.dk/)^54^. For bacterial adhesion, the percentage of covered surface was determined using ImageJ software^55^. At least three image stacks from each of three independent experiments (nine stacks in total) were used for each analysis.

### *Galleria mellonella* virulence assay

*P. aeruginosa* H103 strain, used for infection of *G. mellonella*, were grown in LB supplemented with 1 or 10 µM Epi, for 6 h (late exponential phase). After centrifugation, bacterial cells were washed once in physiological water (NaCl 0.9%) and resuspended to a final concentration of 10^5^ CFU/mL. The size of the inoculum was confirmed by numeration on solid LB. Fifteen larvae were infected with 10 μL of a cell suspension, supplemented or not with Epi, into the hemocoel using a microinjector (KDS100 Legacy, Fisher Scientific) with a sterilized microsyringe^56,57^. Larva were incubated at 37 °C and survival was monitored during 17 h post-infections.

### Cells cultures

The human colon adenocarcinoma Caco-2/TC7 cells were grown in DMEM (Dulbecco’s Modified Eagle Medium) containing 15% of heat-inactivated fetal bovine serum (FBS), and penicillin/streptomycin (100 µg/mL). Cells were cultivated at 37 °C, in 5% CO_2_ - 95% air atmosphere and the medium was regularly changed. For adhesion assays, cells were seeded in 24 wells plates treated for tissue culture and used at confluence.

### Adhesion

Adhesion of *P. aeruginosa* H103 on intestinal Caco-2/TC7 cells was investigated according to Baccouri *et al*.^56^ including some modifications. Bacteria were grown for 6 h in LB supplemented with 1 or 10 µM Epi (late exponential phase), then collected by centrifugation at 8000 x g for 10 min. The pellets were resuspended in DMEM without FBS and antibiotics, added on the confluent intestinal cells at a MOI of 100 bacteria per cell, and allowed to adhere. After 2 h of adhesion, Caco-2/TC7 cells were washed twice with PBS (Phosphate Buffer Saline) to remove planktonic bacteria, and then disrupted with 0.1% triton 100x. The lysates were then diluted and plated on LB agar, to determine the number of adherent bacteria.

### Statistical analysis

Statistical analysis was performed using GraphPad Prism (GraphPad Prism 8.1.2; GraphPad Software, San Diego, California, USA). For the *G. mellonella* killing assay, larvae survival was calculated by the Kaplan-Meier method, and survival differences were tested for significance using the log rank test. In all other assays, *t*-test were used to compare the means within the same set of experiments. Statistical significance was determined at *p* < 0.05.

## Supplementary information

Supplementary information 
