# Supplementary material for: Epinephrine affects motility, and increases adhesion, biofilm and virulence of Pseudomonas aeruginosa H103
Source: Sci Rep. 2019 Dec 27;9:20203. doi: 10.1038/s41598-019-56666-7 (PMC6934790; doi:10.1038/s41598-019-56666-7)
Supplement: Supplementary file 1 — Supplementary information [file 41598_2019_56666_MOESM1_ESM.pdf]

Epinephrine affects motility, and increases adhesion, biofilm and virulence of *Pseudomonas aeruginosa* H103

Mélyssa Cambronel[1], Damien Tortuel[1], Kelly Biaggini[1], Olivier Maillot[1], Laure Taupin[2], Karine Réhel[2], Isabelle Rincé[3], Cécile Muller[3], Julie Hardouin[4], Marc Feuilloley[1], Sophie Rodrigues[1], Nathalie Connil*[1]

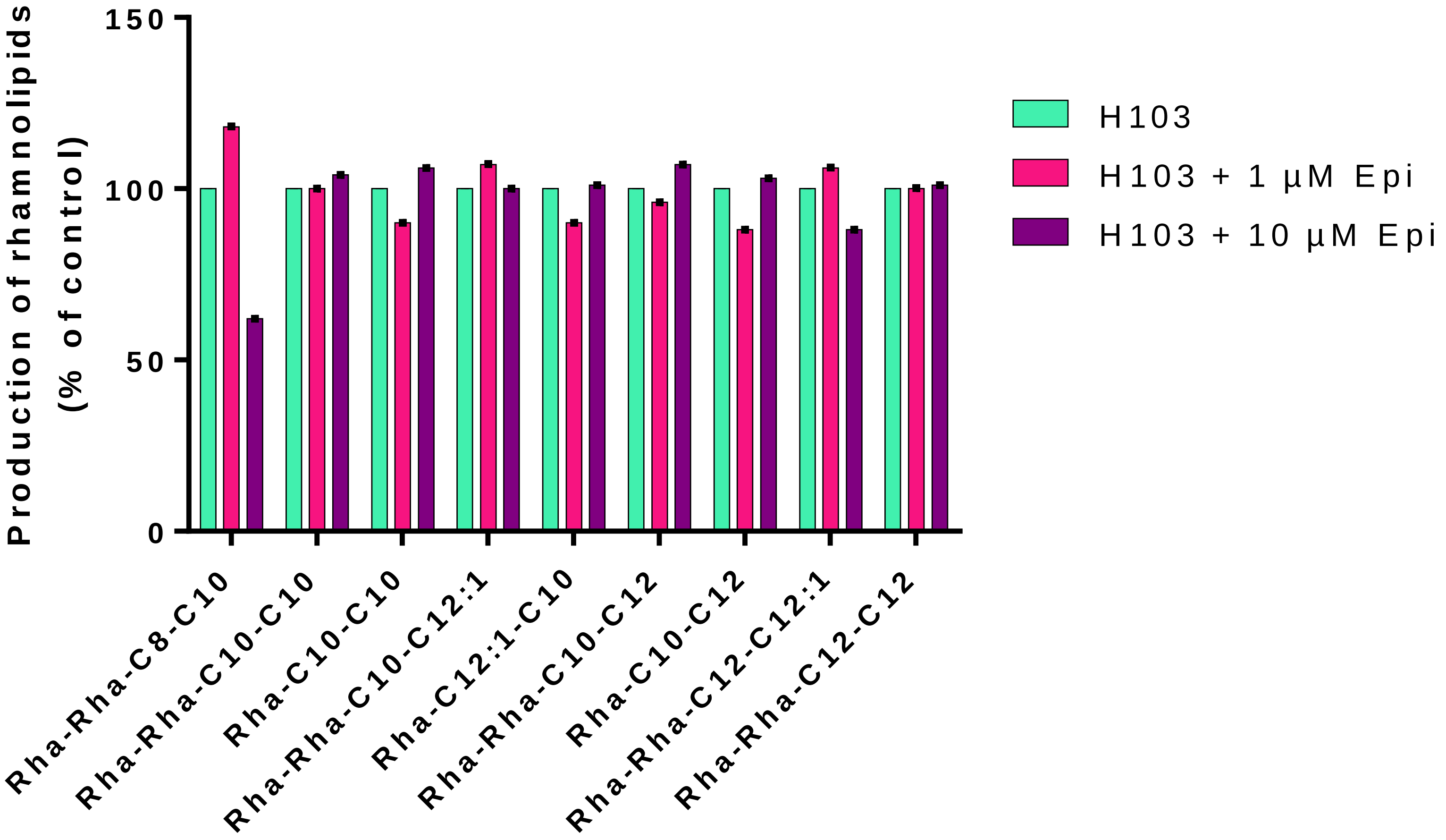

Supplementary data 1. Effect of Epi on rhamnolipids production

Rhamnolipids were extracted after 24 h culture, with or without Epi at 1 and 10 µM and analyzed using mass spectrometry LC-MS. The errors bars indicate the standard error of the mean (SEM) of at least three independent experiments.

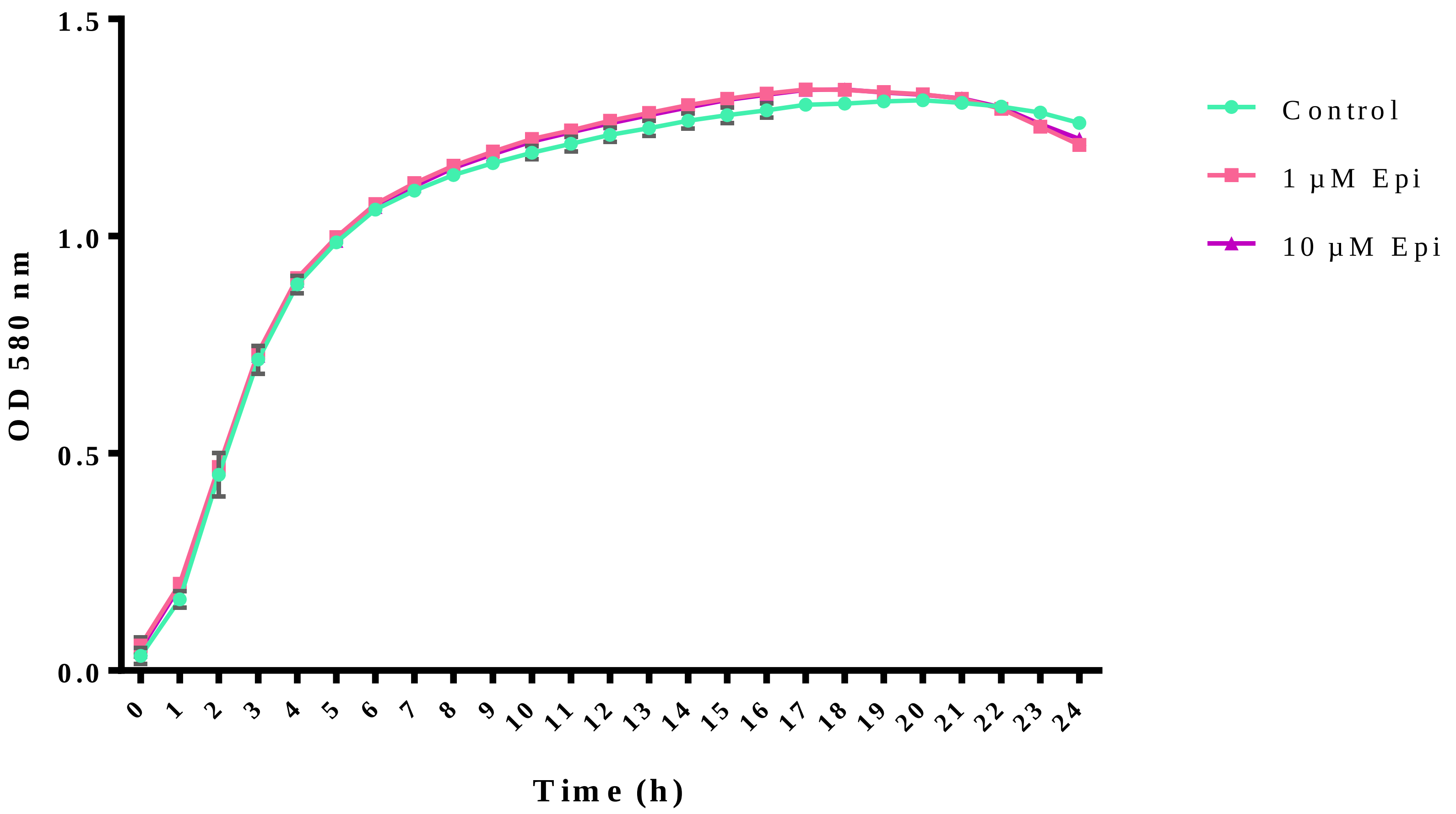

Supplementary data 2. *P. aeruginosa* growth in presence of Epi

P. aeruginosa H103 was grown in LB medium supplemented or not with Epi (1 or 10 µM). The kinetic was performed in a microplate reader and OD_{580} was monitored every 15 min for 24 h at 37°C with orbital shaking.
